# Effects of physical activity on clinical and inflammatory markers in diagnosing multiple myeloma patients

**DOI:** 10.3389/fphys.2022.1094470

**Published:** 2023-01-04

**Authors:** Jiaping Wang, Lixia Sheng, Yanli Lai, Guifang Ouyang, Zhijuan Xu

**Affiliations:** Department of Hematology, Ningbo First Hospital, Ningbo, China

**Keywords:** physical activity, inflammation, multiple myeloma, antibodies, free light chains

## Abstract

Multiple myeloma (MM) is the second most common hematological disorder. Although several drugs have been developed to treat MM, their efficacy is uncertain. In addition, how normal physical activities can decrease inflammatory responses and clinical biomarkers in MM patients needs to be better defined. Therefore, this study evaluated possible clinical and inflammatory markers to determine the early diagnosis of MM during physical activity. This study selected 30 MM patients with normal or no physical activity with ages of >50 years. This study did not require any specific exercise protocols other than noting patients’ daily life activities and considering them as physical activity for 17 days. Then, blood samples were collected to assess clinical and inflammatory markers. Regarding clinical markers, daily life activities in MM patients decreased their LDH, calcium, and β2-microglobulin levels significantly compared to other clinical biomarkers such as creatine and total protein. Further, this study observed no significant differences between daily life activities of MM patients and normal MM patients regarding levels of immunoglobulins except IgM. Furthermore, IL-6 level was significantly increased with the daily life activities of MM patients, suggesting the role of physical activities in increasing anti-inflammatory response along with altering the biochemical profiles including LDH, calcium and β2-microglobulin in MM patients.

## Introduction

Physical activity and exercise have been linked to improving cancer patients’ quality of life in terms of patient outcomes (Spreafico et al., 2021). This may be due to decreases in body composition, systemic inflammation, and oxidative stress and to increased immune function. Studies have shown that exercise increases the immune response in tumors, and the mechanism behind this may be the increased mobilization of immune cells (Sitlinger et al., 2020; Sitlinger et al., 2022). In addition, lower-to-moderate-intensity exercise decreases the risk of cancers and other chronic diseases, especially because it improves physiological and psychological functions among cancer patients (McTiernan et al., 2018; Morishita et al., 2020). This effect has been documented in several meta-analyses. Further, physical activities and exercise impact cellular and bodily functions, which can reduce the recurrence of cancer and increase the survival capacity of patients (Meyerhardt et al., 2006). However, there are no established mechanisms concerning exercise types and cancer types. Further, how exercise influences biochemical inflammatory parameters in cancer patients has been little studied. Therefore, this study aimed to understand how physical activity influences biochemical and inflammatory parameters in multiple myeloma (MM) patients.

Worldwide, cancer has been diagnosed more than ever before in recent years; this may be due to advancements in early detection and effective treatments, which can also increase survival numbers after diagnosis (Loud and Murphy, 2017). Although the treatment strategy has been supportive in increasing survival, the side effects in patients impact their physiological and psychological functions, such as fatigue, pain, and decreased cardiac efficiency. As mentioned above, physical activity and exercise could effectively overcome these side effects. However, designing protocols for MM patients requires great attention as they have weaker immune systems, suggesting that exercise should be prescribed only to those who are in good condition (Nowrousian et al., 2005; Wefelnberg et al., 2022). Furthermore, MM patients with sedentary lifestyles could develop other conditions, such as obesity, cardiac problems, and osteoarthritis, which can accumulate to put the patients in life-threatening conditions. Therefore, performing physical activities can be worthwhile for patients. The present study considers daily life activities as a way to improve MM patients’ quality of life.

## Methods and materials

### Study design

This study divided 30 MM patients into two groups—normal physical activities involved group (*n* = 17) and normal MM patients (*n* = 13)—and followed 17 days of follow-up programs. On the 17th day, they were all asked to donate their blood samples. Prior consent had been obtained from patients, and the analysis had been accepted by the relevant ethics committee.

### Subjects

Every patient seen at the inpatient clinic of the Hematology Department at Ningbo First Hospital from January 2019 to November 2020 was analyzed before treatment. Authorization was acquired from the research ethics board at Ningbo First Hospital (Approval No: 2022RS002). Out of the total 30 patients with MM, 21 females and nine males were selected for blood testing for immunoglobulin profiles, biochemical parameters, and anti-inflammatory markers.

### Inclusion criteria

Newly diagnosed cases of MM of age >55 years, who gave informed consent and fulfilled the diagnostic criteria for MM, were included.

### Exclusion criteria

Patients who did not give consent were excluded.

## Methods

### Free light chain assay and biochemical parameter assays

All the biochemicals and immunoglobulins were measured using commercially available kits (total proteins, calcium, creatine, β2-microglobulin, IgG, IgD, IgM, and free light chains) (Anhui Yi Pu Nuo Kang Biotechnology Co. Ltd.).

### LDH and IL-6 assessments

Serum LDH and IL-6 levels were measured using commercially available kits (Anhui Yi Pu Nuo Kang Biotechnology Co. Ltd.).

### Statistical analysis

Data are expressed as means ± standard deviations (SD). Statistical significance was determined using the non-parametric *t*-test, and significance was set at *p* ≤ 0.05. Graph Pad Prism software version 9 was used for all analyses.

## Results

**FIGURE 1:**
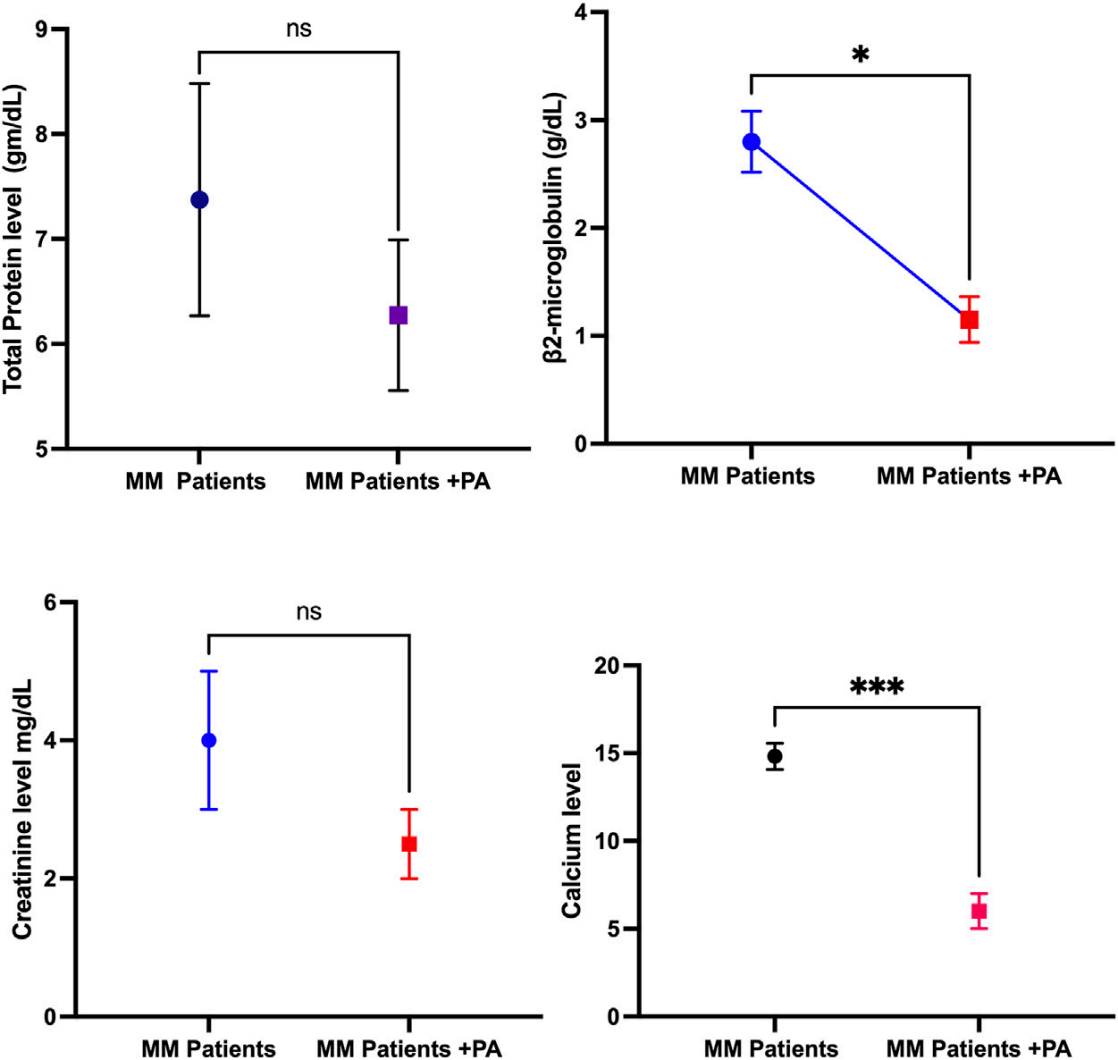
Effects of physical activity with MM on biochemical parameters such as total protein, β2-microglobulin, creatinine level, and calcium level.

Biochemical profiles assist in the early diagnosis of any cancer, and fluctuations in biochemical parameters can impact cancer patients’ health outcomes. Therefore, this study aimed to investigate whether physical activity can influence biochemical parameters such as creatinine, calcium, and total protein levels. This study observed that calcium and β-2 microglobulin levels decreased significantly in MM + PA patients compared to MM patients. This was further reflected in the creatinine levels of the MM + PA patients. However, there were no significant differences observed in total protein levels between MM + PA patients and MM patients (Figure 1).

**FIGURE 2:**
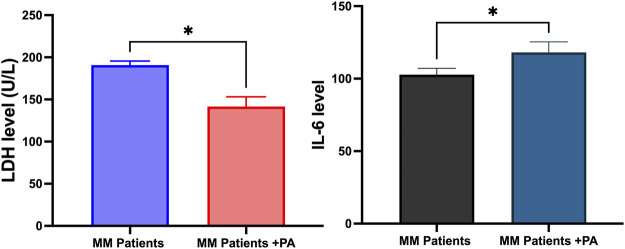
Effects of physical activity with MM on LDH and IL-6 levels.

LDH accumulation indicates the onset of MM on rare occasions. The overall survival rate of MM patients is lower in the LDH accumulation state. This study found that LDH levels decreased significantly in the MM + PA patients compared to the MM patients (Figure 2). Interleukin-6 level is linked with MM severity and tumor cell mass. This study observed that MM + PA patients had increased IL-6 levels compared to MM patients (Figure 2).

**FIGURE 3:**
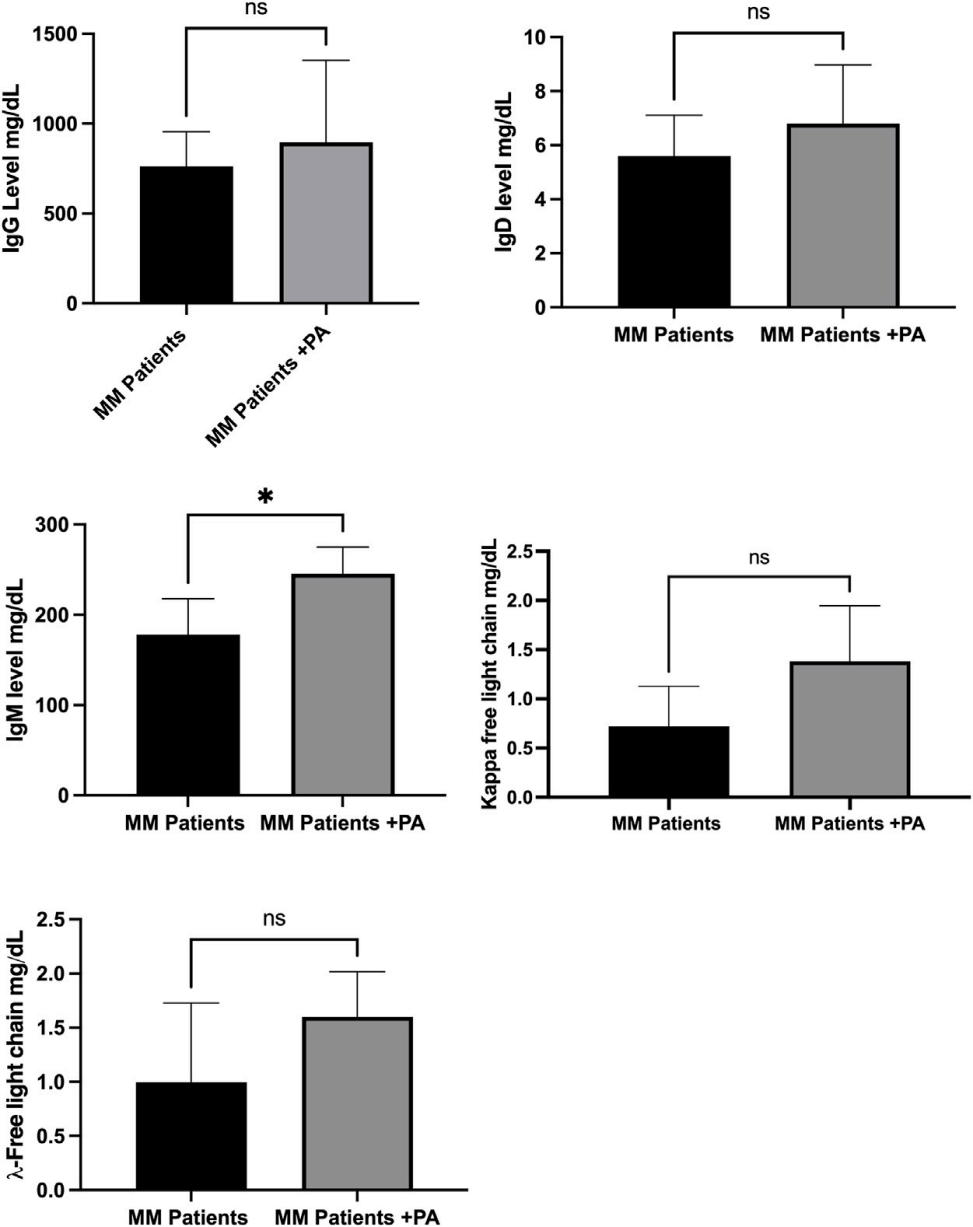
Effects of physical activity with MM patients on immunoglobulins.

Figure 3 shows that MM patients involved in normal physical activities had no changes in levels of antibodies, such as IgD, IgG, kappa, and lambda free light chains compared to MM patients with no activities. However, the IgM level was significantly increased in the MM + PA group compared to the MM group.

## Discussion

Physical activities and moderate exercise have improved cancer patients’ quality of life, including in MM. However, different treatments and their side effects and co-morbidities hinder the prescribing or performing of exercise for gaining the full benefits of exercise in improving cancer patients’ quality of life (Teke et al., 2014). Proper exercise may improve survivorship among patients. LDH level is correlated with MM disease progression. Studies have showed increased LDH in MM onset and disease progression (Barlogie et al., 1989; Terpos et al., 2010). This may be due to increased tumor mass. This study observed that normal physical activities decreased the LDH level relative to the MM patients, suggesting the role of physical activities in decreasing LDH levels. At the same time, daily activities increased IL-6 levels in the MM patients, suggesting the possible effect of physical activities in improving anti-inflammatory response in the MM patients. Studies have linked IL-6 with increasing the pathogenesis of the MM condition by preventing the MM cells from undergoing apoptosis, suggesting that targeting IL-6 could be an effective strategy for reducing tumorigenesis (Gadó et al., 2000). In contrast, other studies have shown that IL-6 activates, proliferates, and increases the survival rates of lymphocytes during the immune response, suggesting a dual face of IL-6 in regulating various signaling pathways in the tumor microenvironment (Fisher et al., 2014). In this way, physical activities reshape the IL-6 pathways from negative to positive effects for improving MM conditions, possibly interfering with oxidative stress pathways.

High calcium levels are linked with MM, possibly due to an increase in bone destruction that can release calcium into the blood. Also, abnormal functions of the parathyroid gland can cause an imbalance of calcium levels in MM patients. This study observed a decrease in calcium levels in the MM + PA group compared to the MM patients. This is also partially reflected by the small decrease in creatine levels in the MM + PA patients compared to the MM patients. Several studies have reported that increased calcium levels increase blood creatinine (Faiman et al., 2011; Martínez Mateu et al., 2011; Cheng et al., 2021). However, this is not reflected in total protein levels. Studies have reported that increased calcium levels do not implicate an increase in total protein levels. This may be due to hypercalcemia with metastatic osteolytic carcinoma; in this scenario, protein levels are normal (Gutman and Gutman, 1936).

The types of MM are categorized according to the production of immunoglobulins, and MM plasma cells can produce monoclonal immunoglobulins, which are inefficient in fighting infection. This study observed that daily life activities had an impact on increasing levels of immunoglobulins such as IgM. Studies have shown that exercise increases immunoglobulin, which may be due to exercise-induced immune activation or immunoglobulin redistribution (Campbell et al., 2016). Similarly, this study shows that daily activities can induce immune activation by increasing IgM levels. However, other immunoglobulins were also increased, suggesting that better differentiation of all these immunoglobulins under physical exercise conditions is required, as they can be produced by both normal and MM cells.

## Conclusion

In conclusion, MM patients involved in normal physical activities had increased immune stimulation, as confirmed by increases in IgM and other immunoglobulins. MM patients also had decreased levels of calcium and creatine, suggesting that daily physical activities may increase quality of life and increase immune stimulation in MM patients. However, this study had some limitations, such as determining the type or duration of physical activity among the patients and minimal sampling, which can obscure the complete effects of exercise and physical activity. Therefore, further studies are warranted to prescribe exercise and physical activities as the main tools to improve the patient’s quality of life.

## Data Availability

The original contributions presented in the study are included in the article and supplementary material, and further inquiries can be directed to the corresponding author.
